# Associations between granulysin and ovarian endometriosis: A 2-sample Mendelian randomization study

**DOI:** 10.1097/MD.0000000000049618

**Published:** 2026-07-17

**Authors:** Baoyu Lai, Meixing Yu, Yunsheng Liao, Guixin Zhou, Liying Liu, Zhuhuan Shan, Jiaying Fan

**Affiliations:** a Department of Obstetrics and Gynecology, Guangzhou Medical University, Guangzhou, China; b Department of Obstetrics and Gynecology, Guangzhou Women and Children’s Medical Center, Guangzhou, China.

**Keywords:** causal inference, endometriosis, granulysin, immune dysfunction, Mendelian randomization analysis

## Abstract

Endometriosis (EMS) is a chronic inflammatory disease defined by the presence of endometrial-like tissue outside the uterine cavity. Ovarian EMS is considered the most prevalent disease phenotype. However, the causal relationship between granulysin and ovarian EMS remains unclear. We investigate the potential causal relationship between granulysin and ovarian EMS using a 2-sample Mendelian randomization analysis. Genome-wide association study data for granulysin and ovarian EMS were obtained from publicly available online databases. A 2-sample Mendelian randomization analysis was conducted using the inverse-variance weighted method. The causal effect was further validated through weighted median and MR-Egger regression analyses, and a leave-one-out sensitivity analysis was performed. The odds ratio and its 95% confidence interval were used to evaluate the causal relationship between granulysin and the risk of ovarian EMS. Our findings suggest a direct causal relationship between granulysin expression and ovarian EMS. The inverse-variance weighted analysis revealed that a 1-standard deviation increase in granulysin was associated with a 10.7% reduction in the risk of ovarian EMS (odd ratio = 0.892, 95% confidence interval: 0.824–0.966, *P* = .004). There may exist a negative causal relationship between granulysin expression and ovarian EMS.

## 1. Introduction

Endometriosis (EMS) refers to a chronic inflammatory disease characterized by the presence of endometrial glands and stroma outside the uterine lining. Affecting approximately 10% to 15% of women of reproductive age globally, EMS is the leading pathologic cause of pelvic pain and infertility, with an incidence rate as high as 50% among infertile women.^[1]^ Ovarian EMS is considered one of the most common phenotypes, accounting for 17% to 55% of cases.^[2]^ Although chronic inflammation and abnormally high estrogen levels are well-known characteristics of EMS, the exact etiology of the disease remains unclear. Factors such as genetics, hormones, environment, and immunity have been identified as contributors. The most widely accepted theory so far for the formation of endometrial lesions is that during menstruation, endometrial cells and tissue fragments are propelled through uterine contractions and attach to pelvic structures, triggering inflammatory reactions, fibrosis, and pain.^[3]^ Activation of immune cells triggers signaling pathways, leading to the release of inflammatory cytokines and subsequent accumulation of various cell types at the site of inflammation.^[4]^ Therefore, the indispensability of immune factors is gradually becoming prominent, which is the key theory that we focus on studying. Research indicates that one of the conditions for the development of endometrial lesions is immune system dysfunction, which affects the expression of specific cytokines.^[5,6]^ In the research of nonspecific immunity, the degree of impaired natural killer (NK) cell activity is positively correlated with the progression of EMS. Defects in NK cell function activate endometrial cells that fail to be cleared and become established in the abdominal cavity, enhancing the adhesion and invasion capabilities of ectopic endometrial tissue and promoting proliferation and angiogenesis of endometrial cells.^[7]^ It has been demonstrated that there is no significant difference in the number of peripheral blood and abdominal NK cells between EMS patients and controls. However, the activity of these cells is significantly reduced.^[8]^ Studies based on the differentiation status of circulating NK cells have revealed a reduction in highly differentiated NK cells in EMS. However, functional assessments of NK cell cytotoxicity post-lesion excision suggest an underlying abnormality in NK cells, as this treatment did not improve their performance.^[9]^ This suggests that reduced NK cell cytotoxicity and decreased numbers of moderately to highly differentiated CD16+ NK cells may be closely related to the pathogenesis of EMS.

Human NK cells comprise approximately 15% of all lymphocytes and are defined by their phenotype through the expression of CD56 and the absence of CD3.^[10]^ The majority (around 90%) of human NK cells are moderately to highly differentiated (CD56dimCD16+), functioning as cytotoxic NK cells. Subsets expressing CD57+ and CD16+ markers possess high cytotoxicity and release cytotoxic granules containing perforin, granzymes, and granulysin (GNLY), exhibiting elevated levels of antibody-dependent cellular cytotoxicity. In contrast, approximately 10% of NK cells are immature (CD56brightCD16−), with the ability to produce high levels of immunomodulatory cytokines such as IFN-γ, TNF-β, and GM-CSF, but with lower cytotoxicity.^[10]^ GNLY is a molecule expressed by human highly differentiated NK cells, exhibiting cytolytic activity against various microorganisms and tumors. Most mammals (excluding rodents) express GNLY, which requires cleavage from a 15 kDa precursor to an activated 9 kDa form. Upon recognition of infected/tumor cells, killer cells release their cytotoxic granule proteins into synapses. In conjunction with perforin, GNLY triggers programmed cell death.^[11]^

To further investigate the relationship between GNLY from active NK cells and the occurrence of EMS, we aim to use Mendelian randomization (MR) analysis to verify the causal relationship.

MR analysis, an emerging data analysis method in epidemiology, utilizes single nucleotide polymorphisms (SNPs) as instrumental variables (IVs) to estimate the causal relationship between exposure and outcome.^[12]^ Compared to traditional observational studies, this method is analogous to randomized controlled trials and less prone to confounding factors and reverse causality.^[13]^ This study aims to evaluate the causal relationship between GNLY and ovarian EMS through 2-sample MR analysis using data from large-scale open-access genome-wide association studies.

## 2. Materials and methods

### 2.1. Data sources

Large-scale genome-wide association study (GWAS) data for exposure and outcome were obtained from the IEU Open GWAS Project database (https://gwas.mrcieu.ac.uk/). The study population was restricted to Europeans to minimize bias from race-related confounding factors. As the data used were publicly available, no additional ethical approval was required. The website was accessed on February 25, 2024.

### 2.2. Selection and processing of SNPs

The 2-sample MR analysis was conducted under the following 3 key assumptions: IVs are strongly associated with the exposure, with *F*-statistics > 10 set as a high threshold; IVs are independent of confounders; and IVs influence the outcome only through the exposure.^[12]^

### 2.3. Genetic variants related to granulysin

GNLY-related SNPs were retrieved from the GWAS summary data of SSGAC, encompassing 31,684 participants of European ancestry. Based on the 3 key assumptions of MR analysis, strongly associated SNPs (*P* < 5 × 10^−8^) were extracted from the GNLY exposure database. SNPs with a linkage disequilibrium coefficient *r*^2^ < 0.001 and genetic distance > 10,000 kb were selected to ensure the independence of each IV SNP and exclude the influence of pleiotropy.^[14]^ To assess whether the selected IVs sufficiently explained GNLY expression, *F*-statistics were calculated to evaluate the strength of the IVs. IVs with *F*-statistics < 10 were considered weak instruments.^[15]^ The *F*-statistic was calculated with the formula: *F*-statistic = *R*^2^ × (N − 2)/(1 − *R*^2^), where *R*^2^ represents the phenotypic variance explained by each genetic variant in the exposure. The *R*^2^ formula is: *R*^2^ = 2 × (β)^2^ × EAF × (1 − EAF)/[2 × (β)^2^ × EAF × (1 − EAF) + 2 × (SE)^2^ × N × EAF × (1 − EAF)].^[16]^ *F* > 10 was set as the high threshold in this study. Ultimately, 11 independent SNPs were selected as IVs related to GNLY expression.

### 2.4. Genetic variants related to ovarian endometriosis

SNPs associated with ovarian EMS were obtained from the FinnGen endometriosis GWAS data, a meta-analysis comprising 3231 cases and 68,969 matched European ancestry controls. For the 11 GNLY-related SNPs, summary data were retrieved from the aforementioned ovarian EMS GWAS data. The exposure and outcome databases were merged for analysis, and all included SNPs were not highly correlated with ovarian EMS (*P* > 5 × 10^−8^).

## 3. Statistical analysis

The inverse-variance weighted method was employed as the primary approach for the 2-sample MR analysis, using SNPs as IVs to validate the causal relationship between exposure and outcome. If *P* < .05, heterogeneity tests and pleiotropy tests would be conducted. The weighted median estimator and MR-Egger regression were used to verify the causal effect between exposure and outcome. Cochran’s *Q* test and funnel plots were utilized to detect heterogeneity. In MR-Egger regression, the MR-Egger intercept was used to test pleiotropy.^[12]^ Data analysis was performed using R 4.3.2 software (R Foundation for Statistical Computing), with the significance level set at α = 0.05.

## 4. Results

We retrieved 11 SNPs associated with GNLY. The *F*-statistics for all IVs were >10, indicating the absence of weak IVs in this study. The 11 SNPs selected in our study, involving 31,684 individuals, were sufficient to yield unbiased causal estimates. Using the inverse-variance weighted (IVW) method for MR analysis, we found that a 1-standard deviation increase in GNLY was associated with a 10.7% reduction in the risk of ovarian EMS (odd ratio = 0.892, 95% confidence interval: 0.824–0.966, *P* = .004). The Cochrane’s *Q* statistic for the IVW method was 9.06, indicating low heterogeneity in the primary IVW results of our study, providing reliability for the causal effect. Both the weighted median estimator analysis and MR-Egger test results showed no statistically significant association between the 2 (*P* > .05; Table 1).

**Table 1:** Causal effect from GNLY to endometriosis of ovary.

Method	Number of SNPs	OR	95% CI	*P*-value	Cochrane’s *Q* statistic
MR-Egger	11	0.89	0.79–0.99	.068	
Weighted median	11	0.87	0.80–0.95	.003	
Inverse-variance weighted	11	0.89	0.82–0.96	.004	9.06
Simple mode	11	0.93	0.73–1.17	.575	
Weighted mode	11	0.87	0.80–0.95	.012	

The table presents the causal effect estimates of GNLY on ovarian endometriosis using 5 MR methods with 11 instrumental SNPs. The odds ratios (OR) and 95% confidence intervals (CI) reflect the change in the risk of ovarian endometriosis per unit increase in genetically predicted GNLY levels. The IVW method (primary analysis) showed a significant negative causal association (OR = 0.89, 95% CI: 0.82–0.96, *P* = .004). No significant heterogeneity was detected (Cochrane’s *Q* = 9.06, *P* > .05).

CI = confidence interval, GNLY = granulysin, IVW = inverse-variance weighted, MR = Mendelian randomization, MR-PRESSO = Mendelian randomization pleiotropy residual sum and outlier, OR = odds ratio, SNP = single nucleotide polymorphisms.

**Figure 1.**
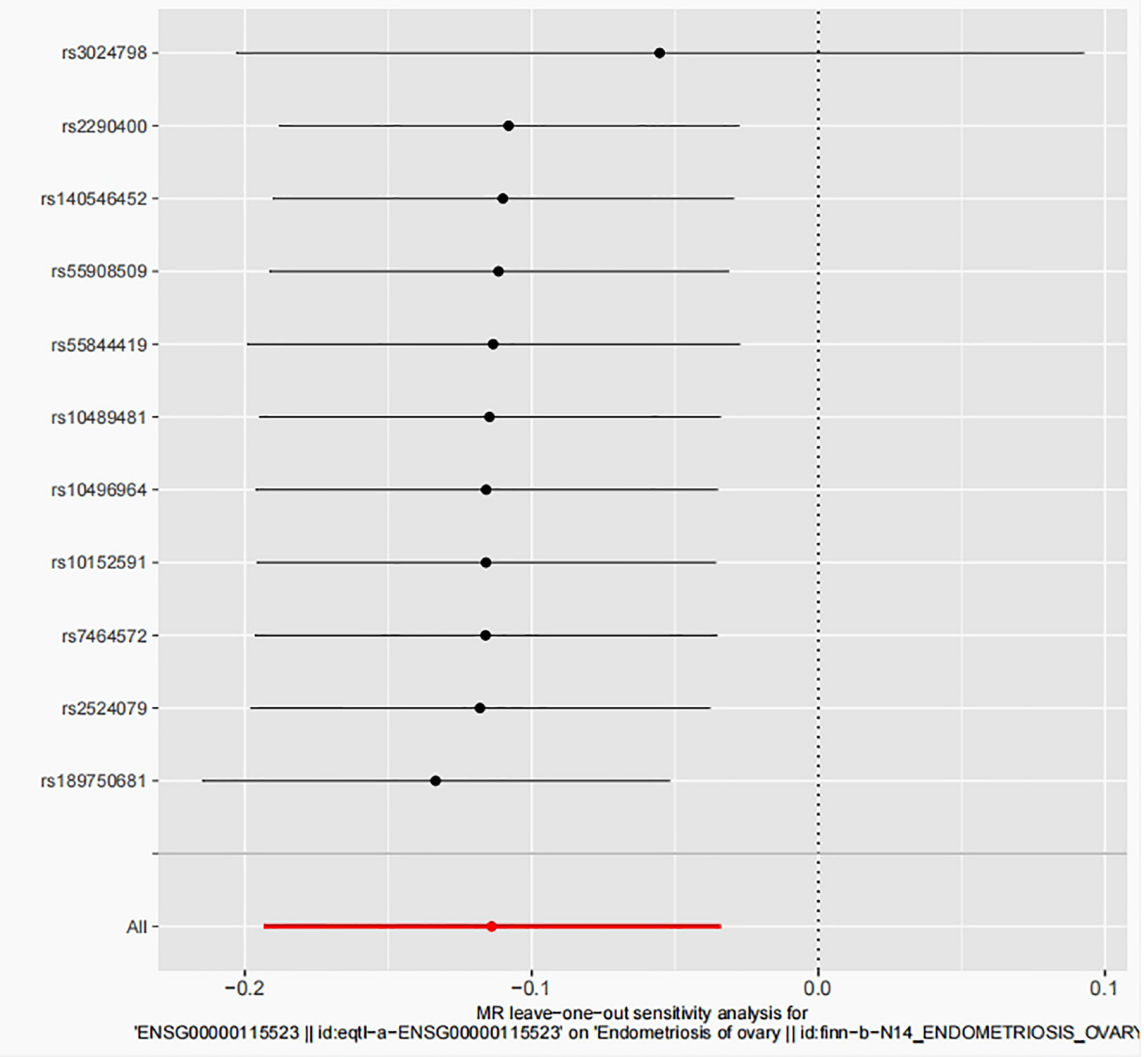
Leave-one-out sensitivity analysis for the causal effect of the GNLY (gene ENSG00000115523) on ovarian endometriosis. Each black dot represents the Mendelian randomization (MR) effect estimate obtained by sequentially removing 1 single nucleotide polymorphism (SNP) from the analysis, with horizontal lines indicating the corresponding 95% confidence intervals. The red line shows the overall MR effect estimate when all SNPs were included in the analysis. All effect estimates remained directionally consistent with the overall effect, and no single SNP was found to unduly influence the results, indicating the robustness of the MR analysis. GNLY = granulysin, MR = Mendelian randomization, SNP = single nucleotide polymorphism.

In sensitivity analysis, the intercept from the MR-Egger regression analysis was 0.116 (*P* = .06), suggesting no significant evidence of overall directional pleiotropy in our causal results (Fig. 1).

**Figure 2.**
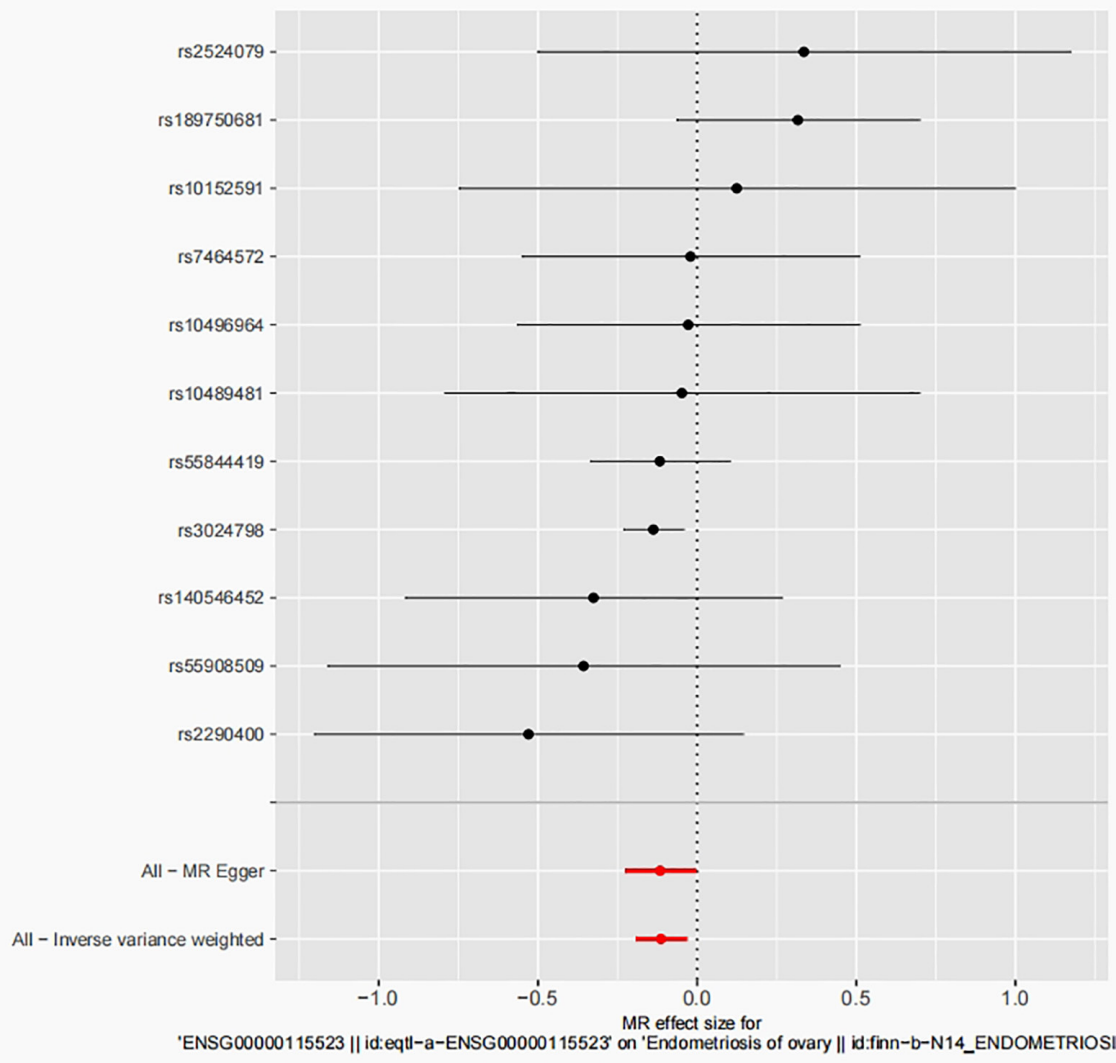
Forest plot of Mendelian randomization (MR) analysis examining the causal effect of GNLY (gene ENSG00000115523) on ovarian endometriosis. Individual SNP-level causal estimates (black dots with 95% CIs) are displayed. The 2 red lines at the bottom represent the pooled results from the 2 MR methods: the upper line shows the MR-Egger regression estimate, and the lower line shows the inverse-variance weighted (IVW) estimate, both with their 95% confidence intervals. The vertical dashed line marks the null effect. Both pooled estimates are located to the left of the null line, with 95% CIs not crossing 0, indicating a significant negative causal association between the gene and ovarian endometriosis. CI = confidence interval, GNLY = granulysin, IVW = inverse-variance weighted, MR = Mendelian randomization, SNP = single nucleotide polymorphism.

**Figure 3.**
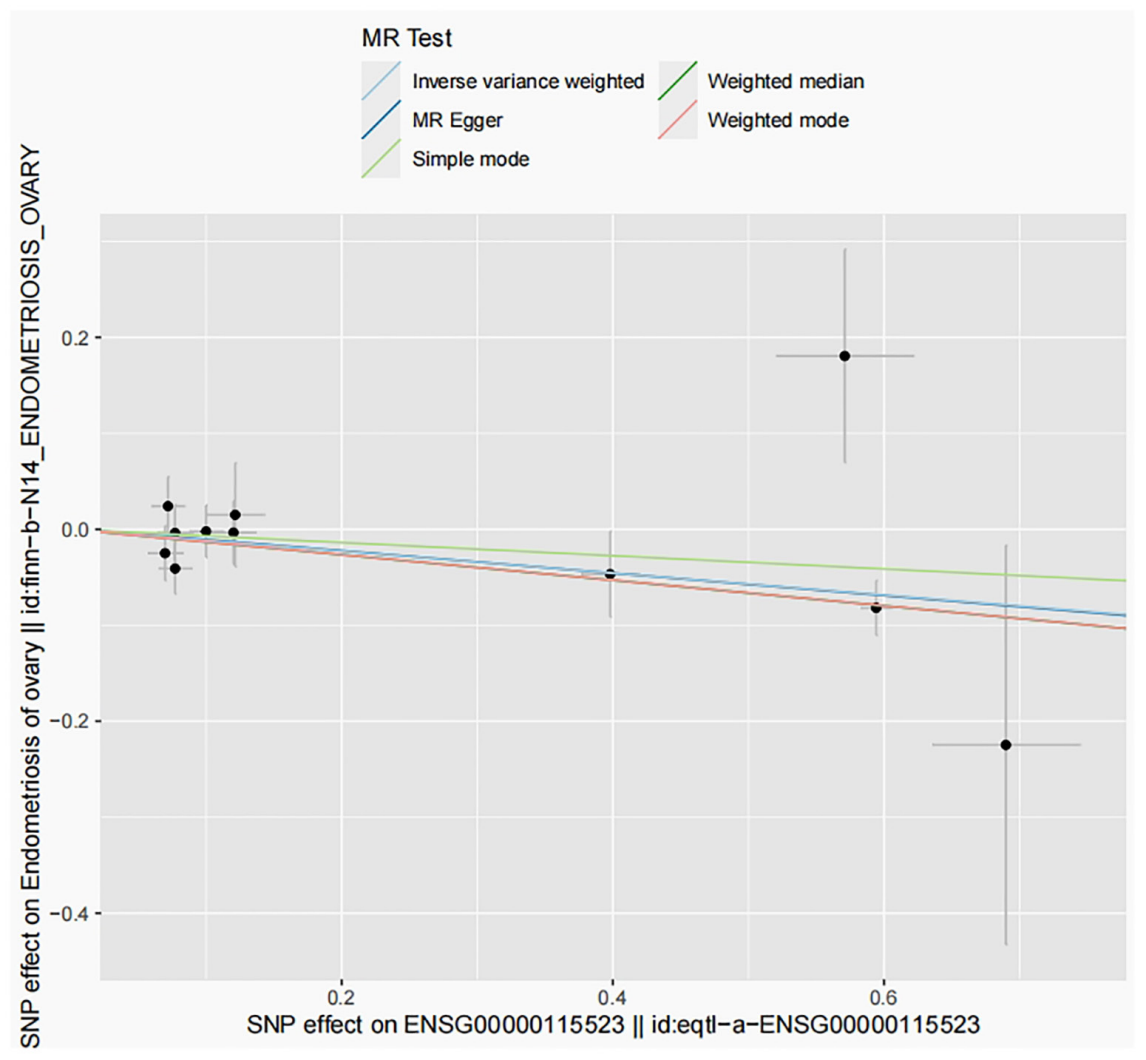
Scatter plot of Mendelian randomization (MR) analysis for the causal effect of gene ENSG00000115523 on ovarian endometriosis. The scatter plot shows the association between the effect of each single nucleotide polymorphism (SNP) on the exposure (GNLY, gene ENSG00000115523, *x*-axis) and its corresponding effect on the outcome (ovarian endometriosis, *y*-axis). Each black dot represents an individual SNP, with error bars indicating the standard error of the effect estimates. Five different MR methods are represented by distinct colored lines: inverse-variance weighted (blue), MR-Egger regression (dark blue), weighted median (green), weighted mode (reddish-brown), and simple mode (light yellow-green). All methods show consistent negative slopes, confirming a robust negative causal association between the gene and ovarian endometriosis. GNLY = granulysin, MR = Mendelian randomization, SNP = single nucleotide polymorphism.

The funnel plot of the 11 selected SNPs exhibited general symmetry, indicating minimal evidence of heterogeneity or pleiotropy in our causal estimates. The consistent direction of causal effects across the 3 tests (IVW, weighted median estimator, and MR-Egger) indicates the robustness of the findings (Figs. 2 and 3).

## 5. Discussion

In this MR study, we have uncovered robust genetic evidence supporting a strong causal relationship between the expression of GNLY and ovarian EMS. Specifically, a 1-standard deviation decrease in GNLY expression is associated with a 10.7% increased risk of developing ovarian EMS.

Previous studies have attempted to investigate the link between immune cell dysfunction and the risk of EMS.^[17]^ Two studies found no significant difference in the number of NK cells in the peripheral blood and abdomen of EMS patients, but a marked reduction in their activity, indicating that NK cell dysfunction is closely related to the development of EMS.^[9]^ Furthermore, EMS is considered a tumor-like disease, and patients with EMS may share similar immune system deficiencies with those suffering from ovarian cancer.^[18]^ In EMS patients, the cytotoxicity of NK cells and T lymphocytes in the peritoneum is reduced, while regulatory T lymphocytes with immunosuppressive effects increase in the ectopic endometrial microenvironment, potentially leading to weakened cellular immunity in the lesions of EMS.^[19]^ Meanwhile, studies based on the differentiation status of circulating NK cells have revealed a reduction in highly differentiated NK cells in EMS.^[9]^

GNLY has been identified as an effector molecule co-localized with perforin in the cytotoxic granules of NK cells and T lymphocytes, capable of inducing cytotoxicity against tumor cells and microbially infected cells. A study^[20]^ found that GNLY expression levels were significantly reduced in cancer patients (*P* < .005), while perforin expression levels remained high. Compared to healthy controls, GNLY levels were significantly lower in patients with progressive tumors (*P* < .0001), accompanied by a decrease in the number of circulating GNLY-positive NK cells. These findings suggest that cancer patients with impaired GNLY expression are in an immunosuppressed state. Recently, a high-throughput single-cell analysis revealed significantly reduced GNLY expression levels in ectopic EMS lesions compared to eutopic endometrial controls, which is generally consistent with our hypothesis and findings.

Our study, leveraging genetic information as an entry point, employed a 2-sample MR analysis to explore the causal relationship between GNLY and ovarian EMS. Although randomized controlled trials (RCTs) are the gold standard for assessing causality, conducting an RCT in this study was impractical for 2 main reasons. First, experimentally restricting GNLY expression in some participants is unavoidable but unethical. Second, EMS primarily occurs during reproductive age, and its diagnosis is often delayed, with an average delay of 8-12 years,^[21]^ implying a long time span between exposure (GNLY expression) and outcome (ovarian EMS). The MR approach is currently the closest method to an RCT, using SNPs to mimic the process of randomly assigning participants with higher and lower GNLY expression levels, similar to RCTs in nonexperimental (observational) settings. The GWAS databases selected for this study were all based on European populations, avoiding biases caused by confounding factors such as environment and ethnicity. Furthermore, the study was conducted using large-sample GWAS datasets, reducing biases due to small sample sizes and resulting in more authentic and reliable findings. However, this study has limitations. First, there are significant differences in genetic susceptibility, environment, lifestyle, and diet between Eastern and Western populations, and this study lacks data from ethnic groups other than Europeans. Future studies should conduct more comprehensive and large-scale research across different ethnic groups. Second, the lack of individual data in this study prevents age or gender subgroup analyses, making it impossible to compare causal effect differences between subgroups.

In conclusion, this study confirms a potential negative causal relationship between GNLY expression levels and ovarian EMS. Low expression levels of GNLY might lead to ovarian EMS. Future research should strive for more comprehensive and large-scale studies with diverse ethnic data. The conclusion of this study provides prospects for further mechanistic research and validation experiments on the role of GNLY as a cause of EMS.

## Acknowledgments

The authors thank the participants of all the consortia mentioned in our study for making data publicly available.

## Author contributions

**Conceptualization:** Baoyu Lai.

**Data curation:** Guixin Zhou.

**Formal analysis:** Meixing Yu.

**Funding acquisition:** Jiaying Fan.

**Investigation:** Liying Liu, Zhuhuan Dan.

**Methodology:** Meixing Yu.

**Project administration:** Jiaying Fan.

**Resources:** Jiaying Fan.

**Software:** Meixing Yu.

**Supervision:** Yunsheng Liao, Jiaying Fan.

**Validation:** Baoyu Lai.

**Visualization:** Meixing Yu.

**Writing – original draft:** Baoyu Lai.

**Writing – review & editing:** Baoyu Lai.

## References

[1] Horne AW Saunders P. SnapShot: endometriosis. Cell. 2019;179:1677. 31951524 10.1016/j.cell.2019.11.033

[2] Chiu CC Hsu TF Jiang LY. Maintenance therapy for preventing endometrioma recurrence after endometriosis resection surgery - a systematic review and network meta-analysis. J Minim Invasive Gynecol. 2022;29:602–12. 35123042 10.1016/j.jmig.2021.11.024

[3] Hsu CF Khine AA Huang HS Chu T-Y. The double engines and single checkpoint theory of endometriosis. Biomedicines. 2022;10:1403. 35740424 10.3390/biomedicines10061403 PMC9219825

[4] Harada T Iwabe T Terakawa N. Role of cytokines in endometriosis. Fertil Steril. 2001;76:1–10. 11438312 10.1016/s0015-0282(01)01816-7

[5] Zhang T De Carolis C Man G. The link between immunity, autoimmunity and endometriosis: a literature update. Autoimmun Rev. 2018;17:945–55. 30107265 10.1016/j.autrev.2018.03.017

[6] Vallve-Juanico J Houshdaran S Giudice LC. The endometrial immune environment of women with endometriosis. Hum Reprod Update. 2019;25:564–91. 31424502 10.1093/humupd/dmz018 PMC6737540

[7] Vinatier D Dufour P Oosterlynck D. Immunological aspects of endometriosis. Hum Reprod Update. 1996;2:371–84. 15717437 10.1093/humupd/2.5.371

[8] Oosterlynck DJ Meuleman C Lacquet FA Waer M Koninckx PR. Flow cytometry analysis of lymphocyte subpopulations in peritoneal fluid of women with endometriosis. Am J Reprod Immunol. 1994;31:25–31. 8166944 10.1111/j.1600-0897.1994.tb00843.x

[9] Oosterlynck DJ Meuleman C Waer M Koninckx PR. CO2-laser excision of endometriosis does not improve the decreased natural killer activity. Acta Obstet Gynecol Scand. 1994;73:333–7. 8160542 10.3109/00016349409015774

[10] Cooper MA Fehniger TA Caligiuri MA. The biology of human natural killer-cell subsets. Trends Immunol. 2001;22:633–40. 11698225 10.1016/s1471-4906(01)02060-9

[11] Clayberger C Krensky AM. Granulysin. Curr Opin Immunol. 2003;15:560–5. 14499265 10.1016/s0952-7915(03)00097-9

[12] Davies NM Holmes MV Davey SG. Reading Mendelian randomisation studies: a guide, glossary, and checklist for clinicians. BMJ. 2018;362:k601. 30002074 10.1136/bmj.k601 PMC6041728

[13] Evans DM Davey SG. Mendelian randomization: new applications in the coming age of hypothesis-free causality. Annu Rev Genomics Hum Genet. 2015;16:327–50. 25939054 10.1146/annurev-genom-090314-050016

[14] Park S Lee S Kim Y. Atrial fibrillation and kidney function: a bidirectional mendelian randomization study. Eur Heart J. 2021;42:2816–23. 34023889 10.1093/eurheartj/ehab291

[15] Burgess S Thompson SG. Interpreting findings from Mendelian randomization using the MR-Egger method. Eur J Epidemiol. 2017;32:377–89. 28527048 10.1007/s10654-017-0255-x PMC5506233

[16] Gogacz M Winkler I Bojarska-Junak A. Increased percentage of Th17 cells in peritoneal fluid is associated with severity of endometriosis. J Reprod Immunol. 2016;117:39–44. 27371900 10.1016/j.jri.2016.04.289

[17] Shim H Chasman DI Smith JD. A multivariate genome-wide association analysis of 10 LDL subfractions, and their response to statin treatment, in 1868 caucasians. PLoS One. 2015;10:e0120758. 25898129 10.1371/journal.pone.0120758 PMC4405269

[18] Lang JH. [Endometriosis and cancer]. Zhonghua Fu Chan Ke Za Zhi. 2019;54:577–81. 31550772 10.3760/cma.j.issn.0529-567x.2019.09.001

[19] Abramiuk M Grywalska E Malkowska P Sierawska O Hrynkiewicz R Niedźwiedzka-Rystwej P. The role of the immune system in the development of endometriosis. Cells. 2022;11:2028. 35805112 10.3390/cells11132028 PMC9265783

[20] Kishi A Takamori Y Ogawa K. Differential expression of granulysin and perforin by NK cells in cancer patients and correlation of impaired granulysin expression with progression of cancer. Cancer Immunol Immunother. 2002;50:604–14. 11807624 10.1007/s002620100228 PMC11032915

[21] Taylor HS Kotlyar AM Flores VA. Endometriosis is a chronic systemic disease: clinical challenges and novel innovations. Lancet. 2021;397:839–52. 33640070 10.1016/S0140-6736(21)00389-5

